# The Revised Version of the *Cyclopædia of Drug Pathogenesy*

**Published:** 1887-01

**Authors:** E. W. Berridge

**Affiliations:** London


### THE REVISED VERSION OF THE CYCLOPÆDIA OF DRUG PATHOGENESY.

E. W. Berridge, M. D., London.

I have now lying before me the third and fourth parts of the above work, including the first volume. It gives me much pleasure to see that in the appendix Dr. Hughes has admitted the validity of my criticisms, and gives a revised version of some of the provings, duly crediting me with the corrections. I have not yet had time to ascertain whether these provings are now completely rendered, but they are evidently far more complete than before. Dr. Hughes admits that *Carbolic acid* was his first error at condensing, and that he had gone farther in that direction than he should. This honorable confession is a good sign for the future, but I must regret that he did not also re-examine the other provings of this drug. Had my papers not been boycotted by the editor of the *Homœopathic World* I should have demonstrated the reliability of many others also.

While the work is evidently now being carried out with more care than before, there is yet room for improvement, and it will not be perfectly satisfactory until all condensation is dropped. I do not mean the condensation of mere *rubiage*, such as some homœopathic as well as allopathic writers indulge in, but all condensation of the expressions with which the symptoms are described. Such condensation often impairs the full force of the meaning, and by rendering the phraseology less graphic, increases the difficulty of remembering it. I purpose therefore to point out a few errors in these two parts. The reader must not, however, consider that these are all—they may be so, or they may not. I can only now examine a few of the provings, leaving the editors to search the rest.

Cannabis indica, No. 3, page 715. In line 4 of proving: "right arm" should be "right upper arm, extending down arm and up to axilla." In line 5 "feet" should be "foot" (misprint in original, copied also by Allen); line 6: after "right side" add "seemed to stop at mesial line;" line 7: "metallic" should be "somewhat metallic;" line 9: "much prolonged" should be "very much prolonged;" line 16, add "he could count his pulse well; it did not seem to him to be beating slowly, though time seemed prolonged; his pulse seemed to him to be full and bounding" (as it really was); line 4 from bottom: for "this" read "this condition of heart." It does not refer to the other symptoms in the same paragraph.

Page 716, line 32, should read "remembered events that had happened, an idea that passed through his mind when a child, as about toys," The remainder of the proving is correct. No. 4, page 716, line 1: a comma should be placed after "short," it was the prover who was "short," not his "dark hair;" line 3: for "one P. M." read "one A. M." (misprint is original); line 1: for "December 7th" read "December 4th" (misprint is original). Page 717, line 2, should be "all kinds of fanciful ideas," otherwise the version is correct; No. 5 is correct; also Nos. 7, 8, and 9 (the latter is my own proving); No. 6, line 4, read "knew he was talking nonsense, but could not stop," otherwise correct.

So far, though this version is not altogether accurate, it is a great improvement on what he gave before. But there are several omissions at which I am much surprised. In *The Organon*, Volume I, page 329, I published some of De Boismont's experiments. These are omitted by Dr. Hughes, who only quotes De Boismont's quotations of Gautier's experiments.

No. 15 is Sherley Hibberd's provings, slightly condensed; for my own part I prefer the more graphic original. But I am at a loss to understand why Dr. Hughes has entirely omitted his second and third provings, which immediately follow the first. They contain some new and important symptoms.

No. 18, Pierce's provings, are very much condensed and many important illusions omitted. On page 727, line 16: "frequent drowsiness" is given in original as "sleepiness, drowsiness." It is also suggested that no mention is made of the effects of a dose of 5M (Fincke), though the symptom produced is almost identical with one produced by the crude drug. At the end of his proving Dr. Pierce gives a short proving on a friend "which bore strong resemblance to dropsy." This I am unable to find either in Hughes or Allen. I can also find no reference in Hughes to that standard work on the subject, *The Hashish Eater*, nor to one recently published here, entitled *Confessions of an English Hashish Eater*. Lastly, in the *Homœopathic World*, 1879–80, I published a part of my collection of experiments, only a portion of which can I find in the *Cyclopædia*. There are several others which I have either in the printed original or in the MS., and as they were all quoted in the "Index to Cases of Poisoning," which I published many years ago in the *Monthly Homœopathic Review*, they surely should have been incorporated here.

Arsenic: Under Arseniuretted hydrogen, Dr. Hughes has omitted two valuable poisonings, Nos. 120 and 194 of my *Pathogenetic Record.*

Arum triphyllum: These provings are so condensed and mutilated as to be almost unrecognizable. For want of space I must refer the reader to the orginals, that he may make his own comparisons. I will only mention here that in Gramm's proving the important symptoms of November 12th are omitted, and others placed under that date which really belong to November 13th!

Asclepias tuberosa: Dr. Saverey's proving is omitted, and Dr. A. C. Jones' proving of *Asclepias incarnata* in *N. A. J. H.* vi, 522.

Baptisia: Dr. Hughes has overlooked Dr. Douglass' corrections in *N. A. J. H.* vii, 228.

Bromium: Under *Kali bromatum* I am unable to find a remarkable case of poisoning reported by Dr. Hincke in the *Organon*, p. 343; also a very suggestive remark by an allopath, which I quoted in *Monthly Homœopathic Review*, 1870, p. 647, that this drug produced symptoms like epilepsy.

Camphor: A reference to Allen will show some provings here omitted. In the *U. S. M. J.*, 1875, December 15th, p. 483–5, some provings of *Camphor* are published by Dr. C. Hering, J. C. Morgan, and Hatch. These I cannot find either in Hughes or Allen. In the *American Observer* (date unrecorded in my notes) is a proving of *Monobromide of Camphor* by Dr. H. W. Taylor, which produced the symptom "appearance of snow flakes falling." This seems also omitted both by Hughes and Allen.

In conclusion, I must strongly protest against some statements by Dr. Hughes which involve not only fatal errors but are inconsistent.

In the *Homœopathic World*, 1886, p. 413, he is reported to have said at the late Convention that the scheme in which our provings have been hitherto arranged, according to the method of Hahnemann, were "unnecessary, misleading, and pernicious." Nevertheless, at p. 14 of the Introduction to the first volume of the *Cyclopædia*, he declares that "the *Materia Medica Pura* and the *Chronic Diseases*, translated into the English tongue, must be regarded as the earlier volumes of the present series. Our index will include them, and they should be possessed by every student of drug pathogenesy." I invite Dr. Hughes to answer hesetwo questions:

(1) If the Hahnemannic schema is "unnecessary, misleading, and pernicious," how is it that it has proved both reliable and indispensable in practice, according to the evidence of all who have faithfully endeavored to follow Hahnemann's rules?

(2) If the evidence of Hahnemann's followers is rejected, and the schema demonstrated, is the opinion of Dr. Hughes, to be "unnecessary, misleading, and pernicious," what will be the use of incorporating the medicines thus arranged in the proposed index to the *Cyclopædia?*

